# P-308. Impactful Implementation of “HAPPI MRSA” - Hospital Onset Pneumonia Prevention and Reduction of Methicillin-Resistant *Staphylococcus aureus* Bloodstream Infections at a Large U.S. Health System

**DOI:** 10.1093/ofid/ofae631.511

**Published:** 2025-01-29

**Authors:** Gemma Rosello, Maribel Ruiz, Dr LaShonda Jones, Rossana Rosa, Dr Susana Flores Villamil, Dr Carol Biggs, Lilian M Abbo

**Affiliations:** Jackson Health System, Miami, Florida; Jackson Health System, Miami, Florida; Jackson Health System, Miami, Florida; Jackson Memorial Hospital, Miami, Florida; Jackson Health System, Miami, Florida; Jackson Health System, Miami, Florida; University of Miami Miller School of Medicine, Jackson Health System, Aventura, FL

## Abstract

**Background:**

Hospital onset (HO) Methicillin-Resistant *Staphylococcus aureus* (MRSA) bloodstream infection is one of the key performance metrics for the CMS Hospital-Acquired Condition (HAC) reduction payment program. MRSA bacteremia is a serious preventable infection associated with increased morbidity and mortality. The goal of our intervention was to implement process changes to reduce MRSA bacteremia and HO pneumonia, improve patient safety, quality of care and decrease healthcare costs associated with treatment.

**Figure 1:**
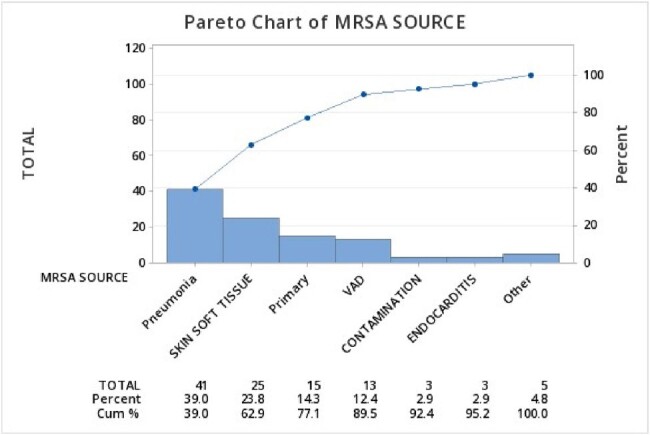
Most Common Sources of All Cases HO MRSA Bacteremia at JHS (Baseline Period: January 2019- September 2021)

**Methods:**

Jackson Health System (JHS) is a 2,289-bed nonprofit academic health system located in Florida, USA. Utilizing a PDSA cycle for process improvement, we implemented a bundle of interventions focused on Hospital Onset Pneumonia Prevention (HAPPI) as the leading cause of MRSA bacteremia across JHS (Figure 1). Planning included focus groups and process mapping to assess barriers to an ideal oral care universal practice (Figure 2). Policies were updated; education and compliance monitoring were incorporated at each hospital. Managers were empowered by hospital leadership to hold staff accountable for each of the metrics within the bundle: universal oral care with kits tailored to independent, dependent, and ventilated patients, universal skin bathing with chlorhexidine gluconate (CHG), nasal decolonization, early mobility, and aspiration precautions.

**Figure 2:**
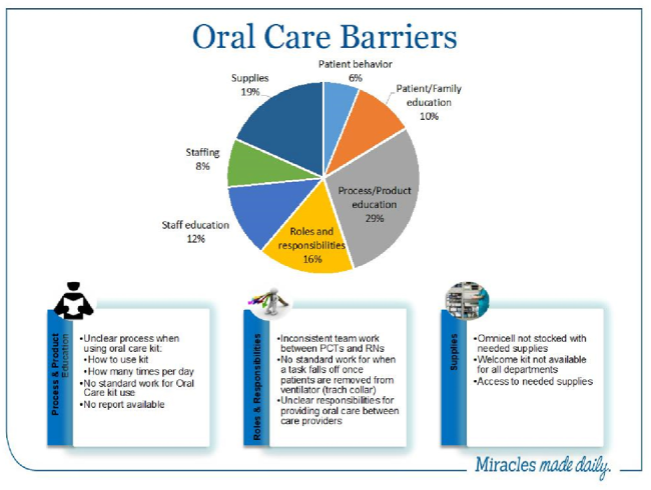
Barriers to Universal Oral Care in Hospitalized Patients at JHS (Focus Groups with Frontline Staff and Infection Prevention Leadership)

**Results:**

During the pre-intervention period Jul 2021-Jun 2022, we identified 46 HO MRSA bacteremias that were secondary to HO pneumonia. System wide education occurred Jul-Dec 2022. During our post-intervention period Jan-Dec 2023, 20 HO MRSA bacteremias were identified that were secondary to HO pneumonia, a 57% reduction (Figure 3). Estimating $60,000 to treat MRSA bacteremia secondary to pneumonia, we identified a $1.5 million dollars in cost savings by preventing 26 infections.

**Figure 3:**
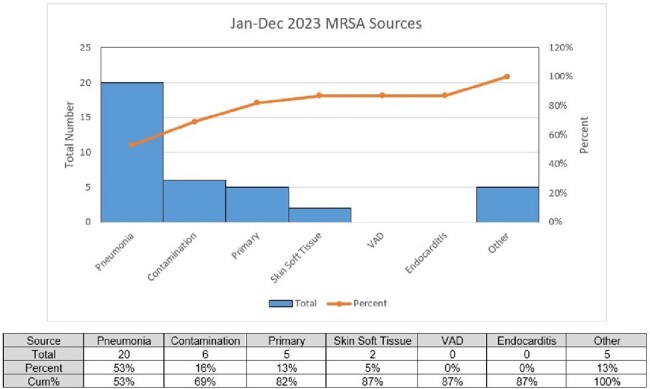
Most Common Sources of All Cases HO MRSA Bacteremia at JHS (Post-Intervention Period: January-December 2023)

**Conclusion:**

We reduced HO MRSA bacteremias and subsequently HO pneumonia after implementing and actively monitoring compliance with a bundle of interventions. Facilities considering pneumonia and MRSA prevention should include strategies that reduce organism bioburden with bathing, nasal decolonization, and oral care along with early mobility and aspiration precautions.

**Disclosures:**

**All Authors**: No reported disclosures

